# Treatment of Fracture of the Maxillæ

**Published:** 1892-12

**Authors:** William Carr

**Affiliations:** New York


### International Dental Journal.

Vol. XIII. December, 1892. No. 12.

### Original Communications.[1]

### TREATMENT OF FRACTURE OF THE MAXILLÆ.[2]

BY DR. WILLIAM CARR, NEW YORK.

Numerous methods and appliances have been devised for treating fractures of the maxillæ, some of which have fallen into disuse, while others are yet employed with varied degrees of success. Among these are bandages, wiring, clamps, and splints,—the latter, in some form, being generally adopted as the most practical.

An article entitled "A New Method for the Treatment of Fracture of the Maxillæ," by Dr. Edward H. Angle, of Minneapolis, was published in the International Dental Journal for June, 1890, in which fractures of the maxillæ were divided into three classes, and for the third class—fractures of edentulous jaws—the following treatment was proposed: "In place of the teeth, small bone-hooks are used, drilling for their reception a suitable cavity on each side of the fracture, comparing in position to the original sockets of the teeth, the same as if the operation of implanting teeth was intended; the cavities thus made need not be so large or deep. They should also be drilled obliquely to correspond to the course taken by the hooks."

[1] The editor and publishers are not responsible for the views of authors of papers published in this department, nor for any claim to novelty, or otherwise, that may be made by them. No papers will be received for this department that have appeared in any other journal published in the country.

[2] Read before the New York Odontological Society, Tuesday evening, October 18, 1892.

This proposed treatment is nearly identical with that mentioned in 1867, by Wheelhouse, of Leeds, England, in case of triple fracture,—jaws not edentulous,—in which he used silver pins instead of bone-hooks suggested by Dr. Angle. Humanity protests against such cruel operations when the interdental or intermaxillary splint affords such rational treatment for these fractures. At the present time, persons with edentulous jaws, even though poor, are rarely without artificial dentures, which may readily be converted into a splint by removing the incisors and canines from the upper denture to afford sufficient space for receiving nourishment; then articulating the upper and lower dentures and firmly uniting them. Such a splint will meet all the requirements of fractures of edentulous jaws, and it may properly be termed an intermaxillary splint. In 1848, Hullihen invented the intermaxillary splint, which was the outgrowth of the interdental splint invented by Desirabode in 1823. The following distinction between the splints is made by Dr. Farrar in the work on "Correction of Irregularities of the Teeth:"

The *interdental* is used to retain the teeth in position after correcting irregularities, and also to retain teeth in original position which, having been knocked out, accidentally or otherwise, have been replaced in their sockets, while the *intermaxillary* is adapted to treatment of fractures of the maxillæ.

Within a few years great improvements have been made in interdental and intermaxillary splints, notably in those made by Dr. Kasson C. Gibson, of New York, who has had an extensive experience in the treatment of fractured maxillæ. He first devised the saw-frame splint, which is a saw-frame similar to those used in dental laboratories and by jewellers: the handle being removed, in its place is screwed a piece of brass or German silver for a chin-piece; the upper portion of the frame is split to within a few lines of the perpendicular portion, to correspond with the jaw and teeth, and to this is attached a vulcanite splint. After successfully using this splint for some time, Dr. Gibson devised another appliance, which he has adopted as fully meeting the requirements of cases which need a metal compress. The following is its description, as given in the *Dental Cosmos* of August, 1890:

"*First.* The compressor (used without alteration for all cases), consisting of a horizontal rod (A) and a vertical rod (B), each about two inches in length, meeting at right angles. Rod B is hollow, and at its upper end is fitted a thumb-screw so adjusted as to hold at any point in it a movable rod (C), thus regulating pressure. Passing horizontally through a bulb at top of rod C is a rod (D)

three inches in length, bent downward at right angles for about an inch. One-half of this lower end is smaller in diameter than the other half, leaving a shoulder. A thumb-screw in the bulb admits of the adjustment of rod D at any angle.

"*Second.* The chin-piece is attached to the compressor by a thumb-screw, so adjusted that it may be pushed backward and forward on the rod A, and at the same time affording a double rotary movement. The chin-piece is made in different forms, each peculiarly adapted to the location of fractures. If fracture is at or near the symphysis, the chin-piece consists simply of a flat piece of brass placed directly under the chin ; if back of the cuspid teeth, involving the molars, it should fit the chin, extending back to the angle of the jaw ; if involving both the angle and the condyle, it must include the entire lower jaw, and extend to the line of the ear. A metal button (E) should be attached on each side of the chin-piece for use in attaching any necessary support. For support nothing surpasses a skull-cap, to each side of which can be attached a piece of elastic reaching to the chin-piece; this in turn can be fastened to the metal button by a ring sewed in the end of the elastic.

"*Third.* The inner steel bands are made of various sizes and shapes to conform, as nearly as possible, to the contour of the jaw, and to the teeth, and are vulcanized in the splint."

This splint enables the patient to masticate freely, and it is efficient, whether the fracture is simple, compound, or complicated. Its use is especially indicated where complication—as necrosis or abscess—is liable to occur. But in cases where the displacement is inward and outward, the wire splint devised in 1876 by Dr. Gunnel E. Hammond, of Paris, and modified by Dr. Gibson, is preferable. The objection to this splint as originally devised was, that in masticating, the ligature securing the splint to the teeth worked below the free margin of the gums, greatly irritating them, and also causing elongation of the teeth. In the modification this objection was overcome by soldering pieces of wire from the buccal to the lingual surface of the splint between the molars and bicuspids, which effectually prevents the splint from settling or irritating the gums. Should the wires between the teeth interfere with proper mastication, this difficulty may be obviated by covering the entire surface with gutta-percha.

In my own practice I have used the appliance known as Williams's Modified Interdental Splint, which is an improvement of the Gunning splint. The principal advantage of using this lies in the fact that the patient is enabled to continue his usual occupation without attracting special attention to his condition, as there are no external appliances connected with it. He cannot masticate, but receives nourishment in a liquid or semi-liquid form.

There is but little difficulty in establishing the correct diagnosis of fracture of the maxillæ, as the following symptoms are usually. present: Severe pains in the effort to open or close the mouth, swelling, crepitus, inflammation, inability to masticate, and marked irregularity of the teeth, with more or less displacement.

Fractures of the maxillæ are caused either by accident or by direct violence; usually by accident when the superior, or both superior and inferior are involved, and by violence when the inferior only is fractured. A noticeable circumstance in connection with these fractures is that the patient rarely applies for treatment for several days succeeding the injury, which, if resulting from violence, is usually inflicted while fighting, when from intoxication or other causes he is not cognizant of the nature of his injuries. He is aware that some of his teeth are loosened, as also that his face and mouth are badly bruised and correspondingly painful, but he does not apply for surgical aid until alarmed by the increased inflammatory condition of the parts.

Fractures of the inferior maxilla may occur at any part of the bone; they are rarely found at the symphysis menti or at the coronoid process. Occasionally the ramus and angle are fractured. Should the condyle be fractured it will usually take place at its neck, resulting from a fall upon the chin or from a blow received at or near the angle. Most frequently fractures occur in the body of the bone in the region of the mental foramen.

Although there may be considerable displacement in fractures of the superior maxilla, yet, if properly treated, serious complications seldom arise, owing to the great vascularity of the parts.

The treatment is identical with that for other fractures,—namely, to bring the parts into apposition and retain them firmly until ossification is complete. When the splint is properly adjusted, speedy union may be secured without deformity of the jaw or irregularity of the teeth. Before taking an impression a careful examination of the parts should be made by passing the finger along the margin of the jaw, to ascertain whether any foreign substance or loose pieces of bone are present. No effort should be made to reduce the fracture before taking an impression, as this is practically useless and inflicts unnecessary pain. Should there be loose or fractured teeth or diseased roots, they should be removed immediately, as their retention might possibly interfere with union.

Also, all exposed pulps should be devitalized, as they might cause considerable suffering during treatment.

When the inferior maxilla only is fractured, an impression of the upper jaw should first be taken in order to secure the confidence of the patient; then take an impression of the lower jaw, using an ordinary lower impression-cup, avoiding the use of too much compound, as only an impression of the teeth is required. For this purpose use S. S. White's or Hood and Reynold's No. 2 Impression Compound as warm as the patient can bear it, in order to prevent unnecessary pain; also, to prevent further displacement of the parts, allow the compound to remain until hard; this will insure a sharp impression.

In making models, mix sulphate of soda or salt with the plaster to give solidity, saw the cast at the point of fracture, and articulate with the cast of the upper jaw; unite them with a few drops of melted wax and immerse the cast of the lower jaw in water, keeping it immersed until thoroughly saturated; then reunite the two parts previously sawed by filling the space with thin plaster. When this has hardened, secure both casts in an articulator. Before waxing, trim the necks of the molars and bicuspids of both casts. This trimming is necessary to secure a tightly-fitting splint, as an impression rarely takes the undercuts of the teeth. Separate the casts by means of the screw at the back of the articulator, leaving a space of about four lines between the incisors. This will leave sufficient room for the patient to receive nourishment. Place a strip of ordinary sheet-wax over the teeth of the lower cast, extending it to the free margin of the gum. Also wax in the same manner the teeth of the upper cast as far forward as the *canines;* next place a roll of soft wax on the grinding surface of the molars and bicuspids, and bring the articulator firmly together; remove the excess of wax, and smooth; then remove the articulator and proceed to invest as far as in an ordinary vulcanite case, taking extra precaution to thoroughly saturate the casts with water, to prevent the formation of bubbles while flasking. After removing the wax, paint the teeth with collodion or liquid silex and cover with tin foil; then pack, having the rubber as soft as possible. Vulcanize for two hours at a temperature ranging from 280° to 300°, in order to secure an elastic splint. Before adjustment, deepen the depression for the molars about one line; drill a small hole on the buccal surface of the splint over the grinding surface of each molar, for the purpose of ascertaining whether, after adjustment, the teeth are in proper position; adjust the splint to the upper jaw, and gently bring the lower jaw with the fractured portion or portions into position until it has passed about two-thirds the length of the teeth. Then with a quick, firm movement bring the parts into place; this movement will, necessarily, be very painful for the patient. After adjustment, pass the finger along the body of the lower jaw, to ascertain whether reduction is comnlete. Next apply the four-tailed or the straight bandage, which should be retained from three to five days, after which, in most cases, it may with safety be removed during the day, but it should be replaced at night until the splint is removed.

The patient should be instructed to keep the mouth thoroughly cleansed, rinsing it morning and evening, first with tepid water, afterwards with equal parts of tepid water and peroxide of hydrogen; at intervals during the day listerine mixed with tepid water, in the proportion of one to three, should be used for the same purpose.

In ordinary cases the splint should be retained three or four weeks, according to the age and physical condition of the patient.

Unless unforseen complications should arise, the application of the splint, combined with thorough cleanliness, will usually be all the treatment required.

The following are a few of the cases treated by this method:

Case I.—Mrs. D., aged twenty-eight, during an altercation with her husband, received a violent blow upon the face, which caused a double fracture at the angle of the jaw. Being poor, she applied to a dispensary for treatment. An effort was made to wire the parts, which was unsuccessful, owing to their bruised, swollen, and inflamed condition. The splint was adjusted ten days afterwards and remained for twenty-one days, when the teeth had occluded perfectly without any deformity of the jaw.

Case II.—A plumber, aged forty. During a strike he did not go out with the Union, and was assaulted while returning from work at night, being struck with what he supposed to be a sand-bag, at the lower portion of the body of the jaw. The next day, upon examination, an oblique fracture was found on the right side between the canine and the first bicuspid. The splint was adjusted, and occlusion was perfect in twenty-three days.

Case III.—John B., a clerk, aged thirty-three, while attempting to spring upon a rapidly-moving street-car, fell and fractured both sides of the inferior maxilla between the lateral incisor and canine on the left, and between the bicuspid and canine on the right. A few hours afterwards the splint was applied and retained for twenty-one days. Perfect occlusion.

CASE IV.—S. B., a student, aged nineteen, while playing football and running, with mouth open, struck the teeth of the superior maxilla against the shoulder of a fellow-student with such force that the superior maxilla and alveolus were fractured. The line of fracture was well defined, on the right side between the canine and bicuspid, and extending on the left between the canine and lateral incisor, the teeth within this line being forced inward, so that the labial surface of the teeth of the superior touched the lingual surface of the teeth of the inferior when the maxillæ were brought together. The upper lip was greatly swollen and bruised. When the teeth had been forced into proper position, the line of fracture could not be felt while passing the finger over it. An impression was taken of the entire upper maxilla, and a vulcanite plate constructed similar to those used for the regulation of the teeth. This was adjusted and retained for twenty-one days, when complete union had taken place.

I have examined the teeth of this patient at intervals during the past six years, and find that none of the pulps of the teeth involved in the fracture are devitalized.

CASE V.—Michael M., a bar-tender, aged forty-six. The inferior maxilla was fractured on both sides during a bar-room fight. The fracture occurred on each side at the point where the second molar had previously been extracted. The splint was adjusted and retained for twenty-one days. Result perfectly satisfactory.

CASE VI.—George A., salesman, aged thirty-three. This fracture was due to a discussion of the "McKinley Bill," a violent blow upon the jaw being the final argument. Upon recovering from the shock, the patient returned home and sent for his family physician, who made an examination and decided that although his face was greatly bruised, he was otherwise uninjured, and directed that an infusion of chamomile leaves be applied. As the patient could masticate only with great difficulty, he consulted his dentist, who sent him to me for treatment five days after the occurrence. Upon examination a fracture was found in the right side of the inferior maxilla, between the lateral incisor and the canine. The splint was adjusted on the sixth day and removed on the twenty-first. Occlusion of the teeth perfect.

CASE VII.—Patrick S., a laborer, aged sixty-five, was injured by a pile of bricks falling upon him. His entire body was greatly bruised. The lower maxilla was fractured on the left side between the first and second molars, and on the right side between the canine and bicuspid; the upper maxilla being fractured between the second and third molars, extending forward between the canine and lateral incisor, and involving the palatine bone. An opening had also been made into the antrum. Saw patient six days after the accident, and found that no effort had been made to reduce the fracture. When the mouth was opened the fractured portion of the upper maxilla rested upon the tongue and the teeth of the lower maxilla, being held only by the mucous membrane of the palatine surface. It was deemed advisable to exsect the displaced portion, which was done, and the mucous membrane carried over the exposed antrum and held firmly by compress. The following day the splint was adjusted. When it was removed, in thirty-five days, there was no displacement nor further trouble with the antrum.

Through the courtesy of W. C. Barrett, M.D., of Buffalo, I am enabled to embody in this paper the following report of a remarkable case of green-stick fracture treated by him:

"I was called upon one afternoon by a prominent and skilful surgeon of my city, who was very earnest in his request that I should at once go to his office. On asking him what was the matter, he informed me that I would find out when I got there. I found four physicians, besides the surgeon, and a boy of about twelve years of age lying under the influence of an anæsthetic, whose head had been run over by a tender of a steam threshing-machine. The physicians and surgeon had been at work over him for nearly four hours, and had not succeeded in getting matters in a satisfactory state. The lower jaw had been fractured in two or three places upon the left side, and the parts had been nicely wired into position. The jaw was under muscular control, but upon an attempt to close it, the whole was deflected considerably to one side, and all efforts to remedy this state of affairs had failed. The surgeon said that he, as well as the physicians, were not ashamed to say that they were at their wits' end, and could not account for the condition. He had proposed calling me in as a specialist, and all had concurred. They therefore wished to put the case in my hands for a diagnosis.

"Upon examination, I found that the fractures upon the left side were well reduced, and held by the wires about the teeth. There was no crepitation upon the right side, nor was there any evidence of luxation. When the jaw dropped to its fullest extent and the muscles thereby relaxed, there was no apparent deflection, but upon closure it was carried to the left.

"It was apparent that a diagnosis could only be arrived at by exclusion. The fact that the jaw was under muscular control showed that there was no dislocation. As there was no crepitation and no digital signs of displacement, there could be no complete fracture. But a more careful examination showed that while the wiring held the parts well together and reduced the fracture, it did not entirely prevent a lateral deflection. This, under the circumstances of the case, could not exist without a corresponding divergence upon the other side. There must, therefore, be an incomplete fracture of the right half of the maxilla. This must be across the body of the bone, anterior to or near the insertion of the internal pterygoid and the masseter muscles of that side. The corresponding muscles of the opposite side, under the undue irritation of the fractured parts, contracted violently, and having no opponents to counteract them, the anterior part of the jaw was, at each such contraction, drawn out of its true plane.

"I accordingly announced my opinion that there was a 'green-stick fracture' of the right half of the inferior maxillary bone. 'What,' said the surgeon, 'a green-stick fracture of a short bone?' 'Why should a green-stick fracture of a short bone in a boy of twelve or fourteen be any more strange than the same condition in the long bone of an old man? And yet we know that is sometimes the case,' was my answer.

"After further examination my diagnosis was accepted, and the patient, at the request of the physicians and surgeon, put in my hands for treatment. He was carried to my office and again anæsthetized. Some common gutta-percha was softened by heat and an impression taken of each jaw, the lower one, of course, with the muscles relaxed. These impressions were trimmed to the proper shape for splints, a hole being made at the symphysis, half of it in each one, through which the boy could be fed. The surface of the gutta-percha splints were then softened by heat and put in place. Then, standing directly at the head of the patient, I grasped the left lower jaw firmly with my left hand, the right being placed against the opposite ramus, and by steady and forcible pressure I closed the jaw, at the same time resisting the tendency to deflection, and thus secured a proper occlusion of the two halves of the gutta-percha splint. The softened surfaces, upon coming in contact, cohered at once, and they were held thus until by a spray of cold water they were hardened. Still holding the whole firmly, the surgeon applied a two-tailed bandage and everything was made secure.

"The next day the inflammation had somewhat subsided, the bandages were tightened, the case dismissed to the care of the regular physician who had accompanied him to the city, and the boy was taken home, some fifty or sixty miles away.

"The bandages were removed in due time, and the physician wrote me that the result was all that could be hoped for, the slight inequality in occlusion of the teeth soon regulating itself, while there were no external marks of the injury whatever that were visible."

